# An Integrative Framework for Healthcare Recommendation Systems: Leveraging the Linear Discriminant Wolf–Convolutional Neural Network (LDW-CNN) Model

**DOI:** 10.3390/diagnostics14222511

**Published:** 2024-11-09

**Authors:** Vedna Sharma, Surender Singh Samant, Tej Singh, Gusztáv Fekete

**Affiliations:** 1 Department of Computer Science, Graphic Era (Deemed to be University), Dehradun 248002, India; vednasharma.cse@geu.ac.in; 2 Savaria Institute of Technology, Faculty of Informatics, Eötvös Loránd University, H-1117 Budapest, Hungary; sht@inf.elte.hu; 3 Department of Material Science and Technology, AUDI Hungaria Faculty of Vehicle Engineering, Széchenyi István University, H-9026 Győr, Hungary

**Keywords:** healthcare, deep learning, linear discriminant analysis, grey wolf optimization, health records

## Abstract

In the evolving healthcare landscape, recommender systems have gained significant importance due to their role in predicting and anticipating a wide range of health-related data for both patients and healthcare professionals. These systems are crucial for delivering precise information while adhering to high standards of quality, reliability, and authentication. **Objectives**: The primary objective of this research is to address the challenge of class imbalance in healthcare recommendation systems. This is achieved by improving the prediction and diagnostic capabilities of these systems through a novel approach that integrates linear discriminant wolf (LDW) with convolutional neural networks (CNNs), forming the LDW-CNN model. **Methods**: The LDW-CNN model incorporates the grey wolf optimizer with linear discriminant analysis to enhance prediction accuracy. The model’s performance is evaluated using multi-disease datasets, covering heart, liver, and kidney diseases. Established error metrics are used to compare the effectiveness of the LDW-CNN model against conventional methods, such as CNNs and multi-level support vector machines (MSVMs). **Results**: The proposed LDW-CNN system demonstrates remarkable accuracy, achieving a rate of 98.1%, which surpasses existing deep learning approaches. In addition, the model improves specificity to 99.18% and sensitivity to 99.008%, outperforming traditional CNN and MSVM techniques in terms of predictive performance. **Conclusions**: The LDW-CNN model emerges as a robust solution for multidisciplinary disease prediction and recommendation, offering superior performance in healthcare recommender systems. Its high accuracy, alongside its improved specificity and sensitivity, positions it as a valuable tool for enhancing prediction and diagnosis across multiple disease domains.

## 1. Introduction

**Figure 1:**
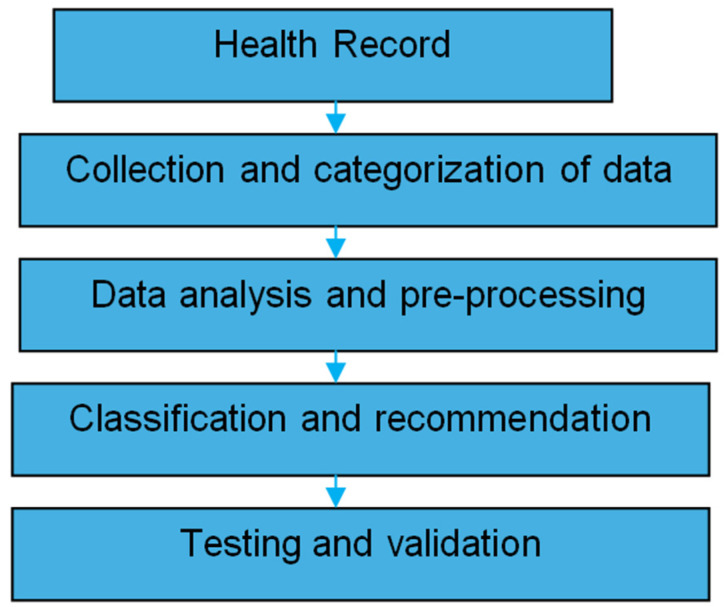
Healthcare recommendation system flowchart [8].

Recommender systems are widely used to provide personalized recommendations to users. They can suggest products for purchase, music for listening, or news articles for reading [1,2]. In the healthcare sector, there is a growing need for precise information about the specific type of treatment required for patients, whether it be emergency care or regular treatment for a particular condition [3]. In the age of abundant online data, recommendation systems have proven effective in addressing the challenge of information overload in diverse industries by providing relevant recommendations [4]. Recommendation systems have proven valuable in healthcare by helping individuals make informed decisions about their well-being. These systems offer personalized technological advice to support health monitoring and improvement. Health recommendation systems can help pinpoint individuals at risk for specific diseases by analyzing their health data and historical trends [4,5,6]. By suggesting preventive measures and early detection strategies, these systems play a role in lowering the incidence and impact of diseases. Machine learning (ML) is a type of statistical analysis that facilitates the creation of modeling techniques for such recommendation systems. ML methods, or classification methods, may take in information, analyze everything statistically, and determine the future based on statistical architectural features [7]. Figure 1 presents a flowchart that outlines the entire process, from data collection to the generation of recommendations [8]. The workflow begins with the acquisition of diverse datasets, which are then meticulously processed and labeled. This pre-processing step is crucial for preparing the data for model training. The labeled data are used to develop and train recommendation models, which are subsequently employed to classify and generate tailored recommendations based on the input data. This structured approach ensures that the recommendation system is accurate and effective in delivering personalized health insights. The choice of algorithms and their performance evaluation are critical for the system’s accuracy and effectiveness in providing relevant health recommendations.

The healthcare industry faces challenges with fragmented data, complicating patient-centered decision-making [9,10,11]. Integrating recommender systems is crucial for enhancing decision-making and improving healthcare quality. Recommender systems are vital for predicting health-related information for patients and doctors [12]. They improve clinical diagnosis and streamline healthcare planning by applying various classification algorithms to healthcare datasets for accurate data classification and practical utility. This paper is structured as follows: Section 2 reviews prior research on recommender systems, summarizing previous studies and methodologies. Section 3 presents the proposed approach, which integrates convolutional neural networks (CNNs) with linear discriminant analysis (LDA) to develop an advanced health recommendation system, further optimized by grey wolf optimization (GWO). Section 4 presents the results and analysis, demonstrating the system’s effectiveness through various performance metrics. Section 5 summarizes the conclusions and explores future opportunities in healthcare recommendation systems.

## 2. Literature Review

In healthcare, substantial research has been dedicated to recommender systems, particularly those that address health-related issues and individual diseases. In this context, the paramount feature of any recommender system is accuracy, aiming to minimize false negatives and positives. Artificial intelligence and machine learning are pivotal in shaping effective healthcare systems [13,14,15,16]. Recommendations are formulated by evaluating the severity of patients’ clinical features, estimating the risk linked to these features and the disease, and calculating the likelihood of the disease occurring. Key focus areas for these systems include precision, user support, and the protection of patient data confidentiality [17]. In healthcare, supervised learning algorithms, which work with annotated data, help predict the presence or likelihood of diseases. Healthcare experts also analyze patients’ medical histories to forecast illnesses. Unsupervised learning techniques, such as clustering and principal component analysis (PCA), group individuals based on symptoms or characteristics. Various algorithms for clustering, classification, natural language processing, logic programming, and semantic technologies are employed in different recommendation scenarios. Given the broad scope of healthcare, obtaining precisely tailored data is often challenging. Deep learning is particularly effective in medical imaging, enhancing diagnostic accuracy, and it is applied to discrete datasets to improve patient care. Previous studies on healthcare recommender systems highlight the need for a comprehensive overview across diverse recommendation scenarios.

In their study, Chinnasamy et al. [8] developed an advanced health recommendation system utilizing a deep learning model that combines the restricted Boltzmann machine and a coevolutionary neural network. This system is designed to mine effectively and analyzes extensive health data to provide personalized medical recommendations and improve decision-making in healthcare. It was developed using Python and its performance was evaluated compared to existing methods. Yoo and Chung [18] designed and developed a recommendation system for healthcare using data mining methods, gathering data through peer-to-peer interactions and adaptive assessment responses. Beyond data processing, the authors proposed a medical care recommendation cellular modem based on mobile services, potentially elevating care standards, lowering costs, and enhancing service quality. K. Subiksha et al. [19] introduced a decentralized medical care framework that leveraged deep learning to establish efficient connections between healthcare databases and services. atural language processing (NLP) methods were employed to convert phrases into queries, utilizing sector-specific conceptual frameworks to identify relevant concepts and relationships within the domain Mudaliar et al. [20] developed a forecasting model to predict medication requirements based on the probability of ailment occurrence, utilizing symptomatic factors and the Naive Bayes algorithm to facilitate the early identification of health issues. Sahoo et al. [21] created an intelligent health recommender system using a deep learning technique that combined the restricted Boltzmann machine and CNNs, showcasing the potential of big data analytics for a robust health recommender engine in telehealth. They applied convolutional neural networks for interpretation and various deep learning libraries for the identification of significant regions, and facilitated computer-aided disease diagnosis or prognosis. In order to estimate mortality risk in patients with idiopathic pulmonary fibrosis, Theodore Armand et al. [22] used five machine learning algorithms (random forest, support vector machine, gradient boosting machine, XGboost, and multi-layer perceptron) to create a soft voting ensemble model. With a prediction error of 0.19, responsiveness of 0.47, sensitivity of 86%, F1-score of 84%, and accuracy of 79.58%, the model performed well. This approach may assist medical professionals in managing patient stratification, lowering mortality risk, and improving patient health.

Sharma and Ahuja [23] proposed a new technique based on data mining for recommending medical care. The main objective of the suggested method was to provide patients with appropriate suggestions based on the present physical situation, as well as health records and restrictions. Individuals were categorized into different groups based solely on their individual characteristics. Then, methods for forecasting the health status of each group were developed. The proposed technique proved effective in providing individuals with appropriate therapies, delivered as reports based on information similarity It also takes personal preferences into account, which are stored on the platform as derived regulations or predicted based on medical history. Mudaliar et al. [20] designed an advanced prediction model to predict disease presence and recommend treatments based on patient data. A unique methodology was proposed for medical diagnosis, which employed a data mining method called the Nave Bayes categorization technique and advice from experts in the forecasted illness. The probability of disease was predicted via detectors of medical emergencies like cardiac output or hypertension, as well as other outwardly detectable indications, including temperature, cough, head pain, and other indications that a person experiences. Sanchez et al. [24] proposed a technique that uses a grey wolf optimizer for achieving optimal data organization and modular architectures in neural networks, specifically for human recognition tasks in biometric measurements. He et al. [25] conducted a study that introduces a cloud-based approach for medical picture segmentation. This technique utilizes multi-feature extraction and interactive fusion to address the constraints imposed by limited local processing capacity. Their system utilizes a Transformer and CNNs to extract both global and local characteristics. Additionally, it incorporates an interactive fusion attention module to enhance the accuracy of segmentation. The efficiency of their technique is demonstrated by the experimental outcomes. 

### Research Gap

The current CNN-based recommendation system has challenges, such as difficulty retaining features, information dropping, and slow computation when used with advanced pooling techniques. A smart healthcare recommendation system provides a wide range of services, which helps them maintain their performance. Such technologies may assist the client and make more accurate patient-centered recommendations. Existing methods for healthcare recommendation systems with research gaps are depicted in Table 1. Many existing models might struggle with accuracy and robustness, especially when dealing with complex and high-dimensional datasets. They may not achieve optimal performance across various healthcare contexts. Existing systems may not effectively integrate diverse analytical techniques, such as by combining LDA with advanced neural networks and optimization methods like GWO. This study aims to create an enhanced hybrid classification model by integrating linear discriminant analysis with CNN and GWO techniques. While CNN architecture has proven effective in recommendation systems, this research proposes a multi-classification system for diseases using the LDW-CNN model. LDA is used in this research to reduce dimensions and preserve data structure, enabling more accurate and efficient feature extraction to overcome the limitations of existing works. GWO optimizes machine learning model hyperparameters, improving the performance of CNN architecture or LDA parameters.

The primary contributions of this research are as follows:

1. Enhanced classification model: The LDA technique in this model extracts discriminative features from high-dimensional data, enhancing classification performance by capturing more meaningful characteristics.
2. Advanced feature extraction and improved class separability: The proposed LDW technique addresses information dropping and slow computation, achieving better class separability for more accurate and reliable recommendations in existing CNN-based recommendation systems.
3. Better model generalization: GWO helps find the best parameters, enhancing accuracy and model generalization in healthcare applications.
4. Comparison: A comparison of the proposed research work with existing CNNs and multi-level support vector machine (MSVM)-based models is presented.

## 3. Methodology

### 3.1. Proposed System

**Figure 2:**
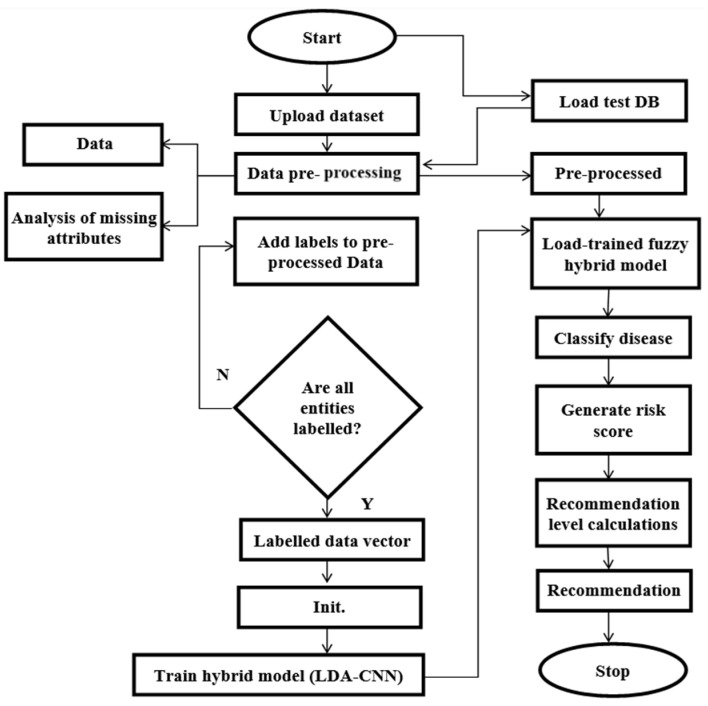
Proposed model.

Our methodology involves utilizing an LDA technique combined with CNN and GWO techniques to design a healthcare recommendation system that can predict the severity of multiple diseases and provide better recommendations. This approach aims to integrate linear discrimination analysis and the wolf optimization technique into healthcare recommendation systems for diseases such as lung and kidney disease. Our primary objective is to build an advanced recommendation system that utilizes patient data to provide accurate disease predictions. By analyzing various health metrics and personal information, our system aims to deliver reliable forecasts that can assist healthcare professionals in diagnosing and managing these conditions effectively. Frequency level is determined by extracting features from pre-processed patient logs using CNN and GWO techniques. The proposed workflow, shown in Figure 2, includes various processing modules within the hybrid recommendation system. Initially, the uploaded dataset goes through data pre-processing modules to eliminate undesirable elements and analyze and handle empty columns. This ensures that the dataset is normalized for training purposes. Subsequently, the pre-processed data are passed through a labeling module, which labels all patient entries. These labels are essential in constructing training models and categorizing results based on dataset entries. The second significant module is dedicated to testing. It helps determine test data labels and generates recommendations by taking into consideration risk scores and disease severity levels. These calculated values are crucial in guiding the recommendation engine to provide tailored suggestions. After all the modules have processed the information and generated results, the performance parameters of the proposed hybrid architecture are computed. This marks the completion of the current thread’s operation.

Our research methodology has several crucial steps, as shown in Figure 2. It includes everything from selecting the dataset to evaluating the defined metrics at the end of the simulation. After that, the proposed process is used to design the research work. Before research analysis, the initial phase involved gathering clinical liver, kidney, and heart datasets from online sources. The data pre-processing phase helped to identify reliable data and eliminate undefined information. During the data pre-processing steps, various methods were applied to remove data cleaning issues, duplicate values, missing information, outliers, etc. To ensure the model was trained effectively, the data was pre-processed in the following steps:

Data Splitting: The dataset was divided into three subsets—60% for training, 20% for validation, and 20% for testing. This split was chosen to ensure a sufficient amount of data for training while keeping a balanced amount for validation and testing to prevent overfitting.

Normalization: All features were normalized to have a mean of 0 and a standard deviation of 1. This step is crucial in ensuring that all features contribute equally to the model’s performance, especially when dealing with features on different scales (e.g., age, blood pressure, and enzyme levels).

Handling Missing Data: Missing values were addressed using mode imputation for categorical features and mean imputation for continuous features. This ensured that incomplete data did not hinder model performance, especially given the importance of complete and accurate data in healthcare prediction tasks.

These pre-processing steps were designed to ensure the robustness of the model and its ability to generalize well across unseen data during testing. The proposed work introduces a feature extraction process using the LDA method. The feature selection was conducted using GWO, and classification was performed using the CNN method to extract reliable features and optimize the feature dimensions of the uploaded clinical dataset.

The hyperparameters for the LDW-CNN model were carefully chosen based on experimental results to optimize the model’s accuracy and generalization capabilities. The final set of hyperparameters, along with the rationales for their selection, is presented in Table 2.

The LDW-CNN model uses a customized CNN to classify heart, liver, and kidney diseases. The dataset is split into the following subsets: 60% for training, 20% for validation, and 20% for testing. The model employs categorical cross-entropy for multi-class classification and is optimized with the Adam optimizer, using an initial learning rate of 0.001. Training runs for 50 epochs with early stopping to prevent overfitting. Additionally, GWO was employed to fine-tune hyperparameters, including learning rate, filter sizes, and dropout rate, by maximizing validation accuracy.

**Figure 3:**
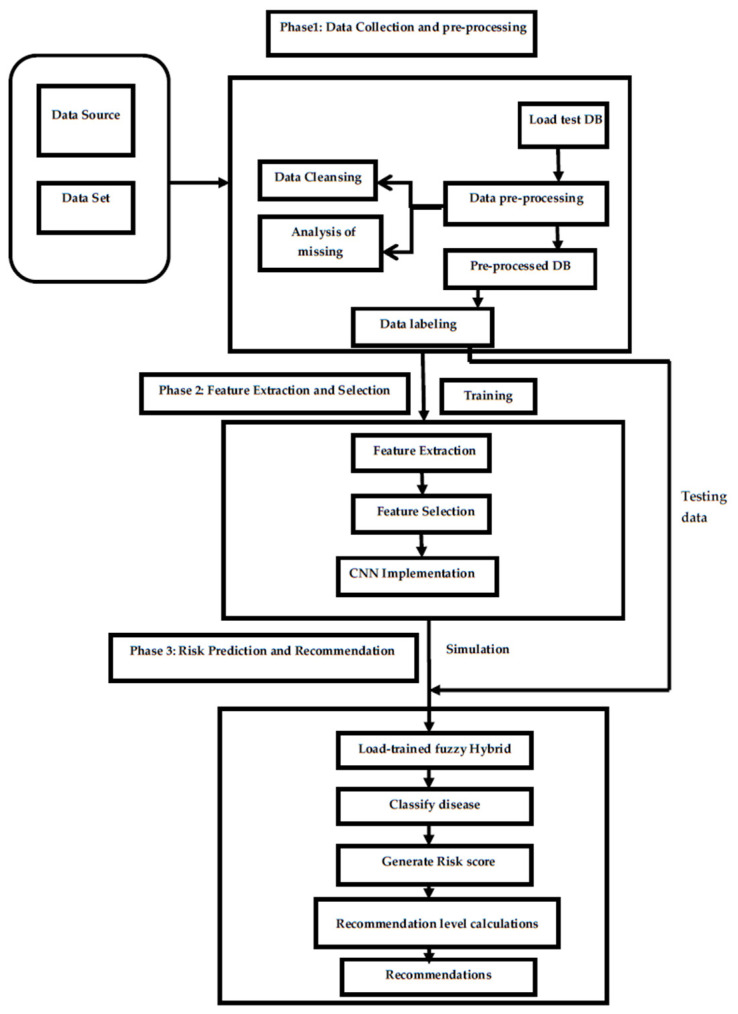
Architecture of the proposed hybrid model.

Pre-processed features from LDA, optimized by GWO, are fed into the CNN, which has two convolutional layers. The first layer has 32 filters (3 × 3) to capture local patterns, and the second has 64 filters (3 × 3) for more complex feature extraction. ReLU activation is used for non-linearity, while Softmax is applied for multi-class classification, providing disease predictions. In our LDW-CNN model, we employ the Softmax activation function in the final layer to handle the multi-class classification problem, in which the model predicts multiple disease types (e.g., heart, liver, kidney diseases). The oftmax function is particularly suitable for this task as it converts the output logits into probabilities, ensuring that the sum of probabilities across all classes equals 1. This allows the model to output the most likely disease prediction with a clear probabilistic interpretation. Other activation functions, such as Sigmoid or ReLU, are less appropriate for multi-class classification, as they do not normalize output probabilities across multiple classes. Therefore, Softmax provides the most interpretable and effective results in this case. The feature-based process worked well with the optimized deep learning method. The architecture of the proposed work is given in Figure 3.

### 3.2. Linear Discrimination Analysis (LDA)

LDA is a classic dimensionality reduction and classification method. It is advantageous when dealing with multi-class classification problems [30,31,32]. LDA seeks to find a linear combination of features that characterizes or separates two or more classes in the dataset. To apply LDA to dimensionality reduction, you can follow the steps outlined below.

1. Evaluate the mean vector ($\mu_{k}$) for each category:

Calculate the mean vector for each category $C_{k}$:

(1) $$\mu_{k}=\frac{1}{N_{k}}\sum_{n\in C_{k}}x_{n}$$

where $N_{k}$ is the number of samples in the category $C_{k}$, and x_n_ represents the feature vector of the n-th sample.

2. Evaluate the within-class and between-class scatter matrices:

Within-class scatter matrix $S_{w}$

(2) $$S_{w}={\sum^{k}}_{k=1}\sum_{n\epsilon c_{k}}\left(x_{n}-\mu_{k}\right){\left(x_{n}-\mu_{k}\right)}^{T}$$

Between-class scatter matrix (S_B_)

(3) $$S_{B}={\sum^{k}}_{k=1}N_{K}\left(\mu_{k}-\mu \right){\left(\mu_{k}-\mu \right)}^{T}$$

3. Evaluate the eigenvalues and eigenvectors for S^−1^_w_S_B_

S^−1^_w_S_B_ = λv (4)

where λ represents the eigenvalues and v represents the eigenvectors.

4. Choose K eigenvectors corresponding to the K largest eigenvalues.

### 3.3. Grey Wolf Optimization (GWO)

GWO plays a significant role in feature selection by effectively navigating the vast search space of potential feature subsets. In this process, GWO utilizes its hierarchical structure, mimicking the hunting behavior of grey wolves, to iteratively update and refine feature subsets based on their performance as evaluated through an objective function. By prioritizing exploration and exploitation, GWO identifies promising subsets of features that optimize the performance of machine learning algorithms while reducing dimensionality [24,33,34,35]. This approach enables GWO to select relevant features efficiently, enhancing the model’s accuracy and generalization while minimizing computational complexity. In crafting the GWO framework, the initial phase involves constructing a hierarchical social model of grey wolves and evaluating the fitness levels of all individuals within the population. GWO’s process is primarily steered by the alpha, beta, and delta wolves, representing the three most adept search agents.

Let x_i_ represent the *i*-th candidate solution, a binary vector indicating the selection status of each feature. The objective function f(x_i)_ evaluates the quality of the feature subset represented by x_i_. GWO iteratively updates the positions of wolves (candidate solutions) using the following equations:

(i) Update alpha, beta, and delta positions

x_alpha_ = x_alpha_ − A⋅D_alpha_ (5)

x_beta =_ x_beta_ − B⋅D_beta_ (6)

x_delta =_ x_delta_ − C⋅D_delta_ (7)

(ii) Update other wolves’ positions

x*_i_* = x_alpha_ − A⋅D_alpha_ (8)

where A, B, and C are coefficient matrices controlling the movement of wolves, and D_alpha_, D_beta_, and D_delta_ represent the distances between the current wolf and the alpha, beta, and delta wolves, respectively. The positions are updated iteratively until a termination criterion is met, optimizing the feature subset represented by the alpha wolf for the given objective function.

The proposed hybrid model uses feature extraction, feature selection, classification, and a recommendation procedure to optimize the error probability and improve the accuracy rate.

The proposed methodology steps are discussed as follows.

Step 1: Dataset upload and text pre-processing

A research analysis dataset is collected from the online site UCI Machine Learning Repository, a dataset of the open-source category with some features. The datasets used in this study cover three primary diseases: heart disease, liver disease, and kidney disease. The details of each dataset, including their key features, are outlined in Table 3.

Heart Disease Dataset: Comprises 303 instances with 13 features, including age, sex, chest pain type, resting blood pressure, serum cholesterol, fasting blood sugar, and maximum heart rate achieved. These features are critical markers commonly associated with cardiovascular health.

Liver Disease Dataset: Contains 583 instances with 10 features, including bilirubin levels, enzyme counts (e.g., alanine aminotransferase), and albumin. Around 70% of the instances represent liver disease patients, while the remaining samples serve as control data.

Kidney Disease Dataset: Consists of 400 instances with a mix of categorical and continuous variables such as blood pressure, serum creatinine, sodium, potassium, hemoglobin, and red blood cell counts. The dataset was split into chronic kidney disease and control samples, allowing the model to assess kidney function accurately.

To ensure data quality and model reliability, missing values were handled through mode imputation for categorical data and mean imputation for continuous variables. Additionally, the data were normalized to achieve a mean of 0 and variance of 1, a crucial step in improving the model’s convergence.

Step 2: Pre-process the data

Pre-processing is an essential step for the efficient representation of the data’s quality. The clinical datasets are pre-processed using methods such as removing missing values, cleaning data from features, etc. The management of missing values is a data pre-processing method used to create smooth datasets. Missing values can be managed in several steps, including disregarding the disappeared value and replacing it with any integer value for that feature. The handling of missing values is indeed crucial, as it can affect model performance, particularly in healthcare applications where accurate data is critical. The performance of the LDW-CNN model heavily relies on accurate data; therefore, rows or columns with missing values are removed if the percentage of missing data is small, and the mode imputation technique is also implemented by replacing missing categorical values with the most frequent category for data pre-processing

Step 3: Feature extraction using LDA

After pre-processing, LDA is applied to clinical disease datasets; LDA can be incredibly efficient for feature extraction, aiming to optimize the dimensionality of the clinical datasets. This is achieved by increasing the between-class and within-class variance ratio in any dataset, ensuring the classes are as different as possible. This method helps verify the features that contribute most significantly to the difference between healthy and diseased cases. The objective of LDA is to solve the generalized eigenvalue problem for S_W_^−1^S_B_, identifying the top KKK eigenvectors that provide the best class separability. These selected features improve the model’s interpretability by focusing on the most informative dimensions of the data.

The steps of the LDA method for feature extraction in a disease dataset generally comprise the following:

- Standardization: entailing that the data is pre-processed to be the same size, i.e., essential for the effective use of LDA.
- Evaluating the scatter matrices: evaluating the within-class and between-class scatter matrices that are important in understanding the division of classes.
- Eigenvalue decomposition: resolving the eigenvalue issue for the matrix derived from the scattered matrices to explore the linear discriminants.
- Choosing linear discriminants: selecting the top linear discriminants depends on the eigenvalue that defines the directions, which increases the separation between classes.
- Prognostic information: transforming the real clinical datasets into the minimum dimensional space shown by the chosen linear discriminants, resulting in the extraction of feature sets.

Step 4: Feature selection using GWO

The GWO algorithm is applied after LDA feature extraction to select the optimal subset of features. The features obtained from LDA are then passed to the GWO for further refinement. GWO treats each feature subset as a candidate solution (wolf) and evaluates their performance based on model accuracy. It is vital in deep learning and data mining, where high-dimensional clinical datasets include noisy, irrelevant feature sets, etc. It aims to optimize data dimensionality and choose reliable feature sets to improve classification performance and optimize the error probability and computation cost. The primary motive of feature selection using the GWO method is to determine a small dataset of feature sets from a specific issue field that reflects a high classification performance [24]. Generally, the feature selection method is used for four reasons:

(i) It creates a simple order for understanding users.
(ii) It takes minimal time to choose reliable features.
(iii) It avoids the dimensionality curse.
(iv) It optimizes overfitting issues.

Step 5: Hybrid LDW-CNN model classification

A new hybrid method has been developed to classify diseases in clinical datasets using a hybrid LDW-CNN algorithm. This method is an improved version of the traditional CNN method, which includes the nature of wolves to enhance the model’s accuracy [36]. The algorithm utilizes a population of candidate solutions called wolves to explore the optimized solution. These wolves are assigned unique locations in a pack, and their rewards are based on their location in the order. This optimizer solves optimization problems with many variables and complex constraints. It is a reliable and effective method that can be applied to various categories of optimization problems. The model was trained and analyzed using a medical dataset of clinical patients with different diseases. The datasets were collected from multiple sources and labeled with the corresponding disease class. The dataset was divided into training, testing, and validation sets, with 60% of the clinical dataset used for training, 20% for testing, and 20% for validation. The hybrid LDW-CNN model is an efficient method for classifying diseases in medical datasets. This model has achieved high accuracy and reduces error probability. The CNN is used to study high-level feature sets from medical data and perform classification tasks based on the features extracted by LDA and optimized by GWO. The hybrid LDW-CNN method improves upon existing clinical database methods using multiple disease classifications. It is more precise and effective compared to the existing methods.

Step 6: Fuzzy hybrid LDW-CNN model recommendation

In this phase, different recommendation systems are used to explore the probability of different levels of health status and give recommendations accordingly. There are specific phases for patients based on their situation, from normal to severe. In this study, we propose a novel approach for disease classification using a hybrid model, referred to as Algorithm 1: Hybrid LDW-CNN. The fuzzy hybrid LDW-CNN model gives the results for all five phases of metric analysis.
**Algorithm 1:** Hybrid LDW-CNN disease classification.**Input**: medical dataset $M_{db}$ **Results**: Different categories of diseases such as liver, kidney, and heart. Upload and read $M_{db}$ ≥ {$M_{db}\}$||$M_{db}$ → medical dataset with features x1, x2, …, xn and class labels Applied pre-process $M_{db}$ data Data normalize [mean of 0 and variance of 1] Split data into train and test datasets Apply the LDA method for dimensionality reduction Evaluate the mean vector (Mv) for each category Evaluate within-class (WC) and between-class (BC) scatter matrix Evaluate E and V (eigenvectors) for WC^-1*BC Choose “K” eigenvectors (V) connected with the maximum E to form a novel feature_space. Initialize GWO metrics Define agents or wolves, α, β, δ, and ω (Alpa, beta, delta, and omega). Set the maxima_number of epochs Define the Objective function (OF): to optimize CNN metrics. Optimized CNN metrics using the GWO method For each epoch: Evaluate the fitness of each wolf using the defined OF Update locations of wolves depend on the best_sol (α, β, δ) Last, until maxima_epochs are reached/convergence criteria are met. The best metrics explored are utilized for CNN method configuration. {$M_{db}$} training_dataset ≥ {$M_{db}\}\;$[0:M] {$M_{db}\}$ testing_dataset ≥ {$M_{db}\}$ [M++: N] CNN_Model_ = intialize_CNN_ (CNN, size ([H, Wt.)])||H = hidden; Wt = Weight Set factors [F, PD, ST]||F = filter; PD = Padding; ST = Stride. Define CNN_Model_ comprising C_Layer_ (CNN_Model_, F, PD, ST, Sig (M)); F_set_ = [[N + 2 P-K] /ST] + 1] Define Pooling_Layer_ (CNN_Model_, subsampling category, and rate) as L||L (labels) = 0,1,2 Set classifier (CNN_Model)_ For init j in 0 L do If (j==L.size) then Classifier = (CNN_Model_, L). End if End for D_type_ = (classifier, {$M_{db}\}$, testing_dataset. Begin F_set_||F_set=_ Fuzzy set Add principles_set R_ec_ to F_set_ and collect||R_ec_ = Recommend Load C_data_||C_data_ = Classify data Load hybrid LDW-CNN model as per C_data_ Create Prob for C_data_||Prob = Probability If Prob > 0 and Prob ≤ 0.5 R_ec_1 = Patient required exercise If Prob > 0.5 and Prob ≤ 1 R_ec_ 2 = Patient required to visit a doctor If Prob ≥ 1 R_ec_ 3 = Patient required to visit a doctor if Prob < 0 R_ec_ 1 = No R_ec_ End if Evaluate performance metrics such as Accuracy, Precision, Recall, etc. Exit

In this study, we experiment on the proposed CNN-GWO-based LDA algorithm with a hybrid heart, kidney, and liver dataset. This experimental research seeks to forecast and offer patient suggestions using data analysis. This study outlines a three-stage process for gauging disease severity: categorization, projection, and guidance.

### 3.4. Performance Metrics

Various validation measures are employed to assess the effectiveness of the proposed method, including measures of accuracy, sensitivity, mean square error (MSE), and specificity. To ensure the robustness and generalizability of the LDW-CNN model, 10-fold cross-validation was employed. This technique divides the dataset into 10 equal parts, using 9 folds for training and 1 fold for validation, rotating the folds in each iteration. The average performance across all 10 iterations provides a more reliable estimate of the model’s accuracy, precision, recall, and other metrics, mitigating the risk of bias from a single train-test split. Accuracy gauges the overall correctness of classifications, while sensitivity measures the method’s ability to identify positive cases correctly. MSE evaluates the average squared difference between the predicted and actual values, reflecting prediction accuracy [29]. Specificity assesses how well the method identifies negative instances. Results are categorized as true positive (TP), true negative (TN), false negative (FN), and false positive (FP). An actual positive result confirms that the method correctly detects the presence of a condition in a patient.

### 3.5. Dataset Availability

Table 3 outlines the health attributes within the datasets, categorizing them by the anatomical regions they pertain to: heart, liver, and kidneys. The heart disease dataset contains 303 records, with each instance representing a patient and their related features, such as age, blood pressure, cholesterol levels, etc. The dataset is split into five categories based on the severity of heart disease. For heart disease, attributes include age, sex, chest pain type, resting blood pressure, serum cholesterol, fasting blood sugar, resting electrocardiographic results, maximum heart rate achieved, exercise-induced angina, and ST depression. The liver dataset contains features such as bilirubin levels and enzyme counts. Around 70% of the instances correspond to patients diagnosed with liver disease, with the rest being control samples. For liver disease, attributes include age, gender, total bilirubin, direct bilirubin, alkaline phosphatase, alanine aminotransferase, aspartate aminotransferase, total proteins, albumin, and the albumin/globulin ratio. The kidney disease dataset consists of 400 instances, and this dataset includes both categorical and continuous features relevant to kidney function. Approximately 40% of the samples indicate chronic kidney disease, while the remaining samples represent healthy or less severe conditions. For kidney disease, attributes include age, blood pressure, specific gravity, albumin, sugar, red blood cells, pus cell clumps, bacteria, blood urea, serum creatinine, sodium, potassium, hemoglobin, packed cell volume, white blood cell count, red blood cell count, hypertension, diabetes mellitus, coronary artery disease, appetite, pedal edema, anemia, and class. This focus allows for a detailed analysis of clinical features related to each condition, improving the accuracy and effectiveness of the health recommendations generated. The three datasets used in this research work are the Heart Disease Dataset [37], the Chronic_Kidney_Disease Dataset [38], and the ILPD (Indian Liver Patient Dataset) [39].

Liver: The liver is a vital organ involved in food digestion. Key indicators that can be used to evaluate liver health include aspartate transaminase (AST) and alanine transaminase (ALT). Generally, these enzyme levels are low under normal conditions [40]. The critical parameters for maintaining a healthy liver are detailed in Table 4.

Kidney: Key parameters for evaluating kidney health status are serum and potassium levels. The acceptable ranges for these parameters indicative of healthy kidney function are outlined in Table 5.

Heart: This research explores the essential role of the human heart in maintaining systemic blood circulation, highlighting how various factors influence this function. It explicitly examines two key biomarkers—cholesterol levels and blood pressure readings—closely linked to cardiovascular health [41]. By analyzing cholesterol levels, this study aims to evaluate the severity of heart-related disorders, while blood pressure measurements are used to assess the associated risk. Table 6 offers a comprehensive overview of cholesterol and blood pressure ranges, detailing their significance and implications for cardiovascular health.

## 4. Results and Discussion

### 4.1. Analysis of Results

**Figure 4:**
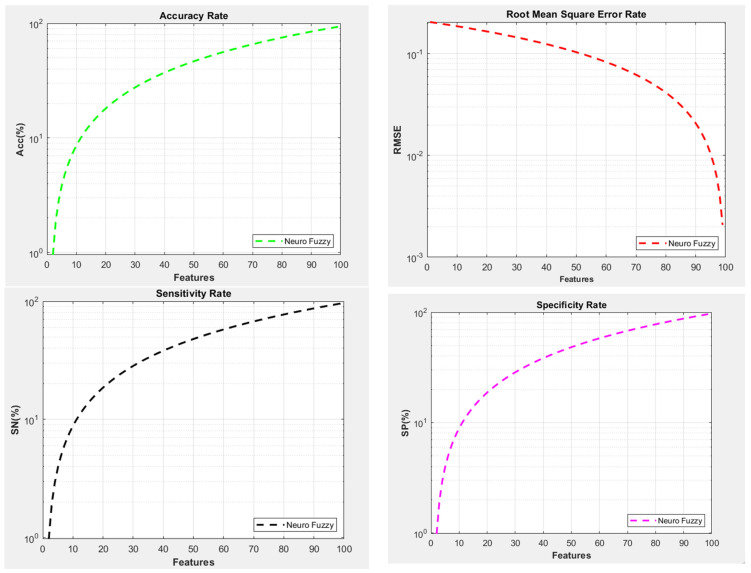
Parameters graphs with Neuro-fuzzy model.

The processing architecture is divided into multiple sub-modules that use different techniques to process data and display the results accordingly. The pre-processing phase includes filters such as data type changes, the placement of unique number patterns in string data, decision-making based on null values, and more. Once all the data are uploaded, they are stored in terms of different variables based on dependencies. The pre-processed data are used in a 4D matrix and are loaded as training and testing data, along with the training and target labels. The collected data are processed through various layers of the CNN, along with the training labels, and the model is then stored in MAT files. These files are database architectures from MATLAB R2018a used to store data in object form for later processing. CNN classification occurs when the model is trained and has stable validation accuracy. The classification process loads the test data from the MAT database and makes predictions using a trained model. After the testing process is complete, the algorithm generates message boxes containing the status of the uploaded sample. The sample is classified into three categories: liver, kidney, and heart. Each category has positive and negative values in terms of Yes and No. The classified data is stored in a vector that is loaded under the recommendation module. The recommendation module uses a fuzzy type-2 model to find similarities and provide recommendations based on the reported parameters of the user’s health. It offers various recommendations based on the calculated probabilities, ranging from no recommendation to a critical situation requiring a doctor’s visit. The CNN model used in this execution performs with 94.2% accuracy, an RMSE (root mean square error) of 0.2057, sensitivity of 96.85, and specificity of 97.43. These parameters determine the model’s performance and demonstrate accuracy when working with different test module sets. In Figure 4, RMSE measures the difference between predicted and actual samples. A lower RMSE indicates higher accuracy.

The existing neuro-fuzzy model had an RMSE of 0.2057. Accuracy is another important factor in evaluating trained models. The higher the accuracy, the better the model is at classification and recommendation. The existing neuro-fuzzy approach had an overall accuracy of 94.2% and depended on positive and negative samples in the given categories. Specificity is a part of accuracy that measures the model’s ability to classify negative samples in the dataset. A higher specificity indicates better performance. The neuro-fuzzy model had a specificity of 97.43. Sensitivity, also known as true positive rate, is related to the model’s ability to classify positive samples in the dataset. A higher sensitivity indicates better performance. The neuro-fuzzy model had a sensitivity of 97.43. Data are collected from the local repository and pre-processed with various filters to generate a normalized dataset for the model. The data are then divided into training and testing sets with associated labels. The model is trained with the training set and stored in the MAT database for use in further multi-level training modules.

**Figure 5:**
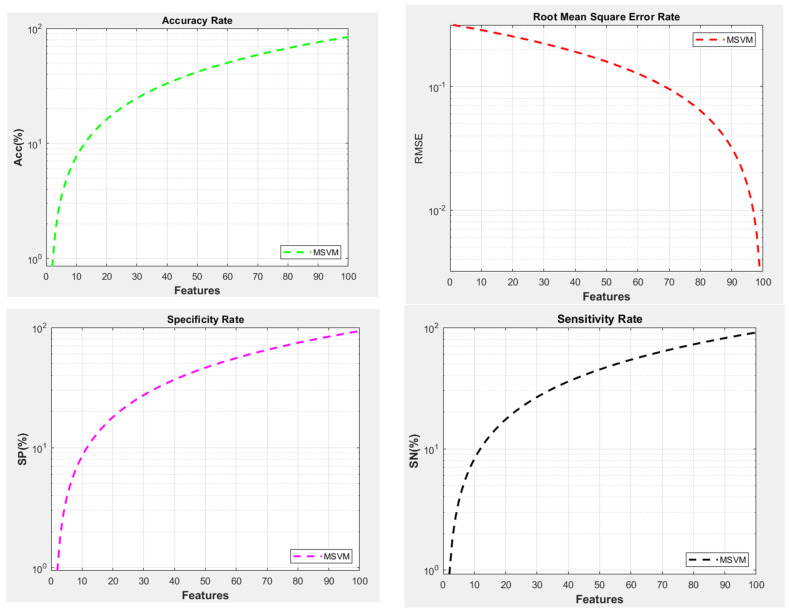
Performance—multi-level graphs.

Multi-level classification is a technique that involves combining multiple binary-based trained models to create a more advanced model. This advanced model is then used to classify data in the test phase. The classified data are then sent to the recommendation phase and the database location so the recommender module can access said data. The multi-level classification method processes similar categories and provides results based on its calculations, like neuro-fuzzy. The recommender module uses a statistical engine to evaluate the calculated score for a particular category. It can recommend no action or suggest a critical situation where the user should visit a doctor. The model’s performance is evaluated at the end of the execution in terms of accuracy, specificity, sensitivity, and RMSE. These parameters provide insight into the classification and recommendation quality of the model. The calculated metrics show an accuracy of 84.3%, an RMSE of 0.3143, a sensitivity of 90.95%, and a specificity of 93.35%. A multi-level processing architecture is used to evaluate the performance of a recommender system. RMSE performance is shown in Figure 5 to be 0.3143. This figure indicates that RMSE performance needs to be optimized as it affects the accuracy rate and degrades the overall performance. RMSE performance is calculated with 100 features at the X level. The accuracy of the multi-level recommender system is also shown in Figure 5. The calculation is performed over various samples, and it calculates the average performance of the test set. The multi-level recommender system shows an accuracy of 84.3%. This demonstrates the system’s efficiency, which is lower than that of the fuzzy CNN architecture on the same test set. Apart from accuracy, specificity is another critical parameter, as shown in Figure 5. Specificity is a part of accuracy that measures the negative samples within the dataset. This parameter helps in finding the classified negative samples over actual negative samples within the dataset. Specificity should be high so the model can evaluate negative samples accurately within the given set for testing. The multi-level recommender model showed a specificity of 93.35. Figure 5 illustrates that the sensitivity parameter, the actual positive rate, plays a crucial role in classifier performance. Like specificity, sensitivity is determined by the number of positive samples the classifier correctly identifies. A high sensitivity is necessary for improving the classifier’s performance. During the test phase, the multi-level recommender model showcased a specificity of 90.95% for the given dataset.

**Figure 6:**
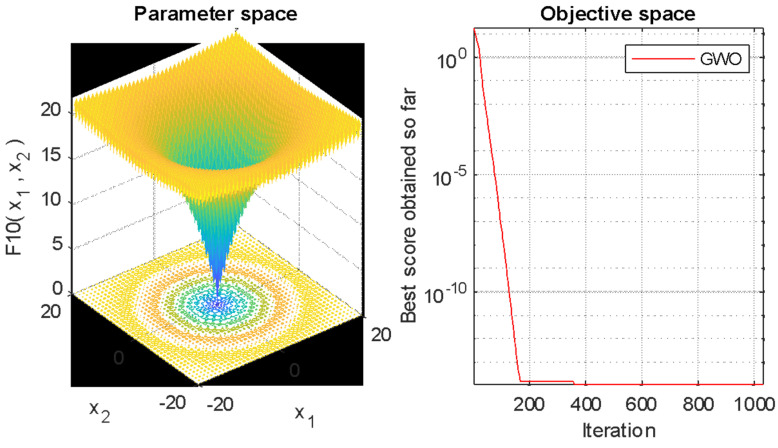
LDW-CNN optimization phase.

The hybrid LDW-CNN model is an optimization-based learning architecture that extracts features from a dataset and optimizes them to minimize error probability and increase accuracy. Once all the modules of the proposed architecture are executed, the performance parameter will be evaluated and displayed in the designated section of the screen. The optimization of the LDW-CNN model allows for the calculation of the best score toward a particular iteration in which the data is processed, as shown in Figure 6. It reduces the error probability and enhances the quality of features calculated from the given data. The process of optimization builds a reduced data matrix and provides a high-quality feature matrix after processing the given iterations. The proposed LDW-CNN architecture is trained using an optimal feature process at the optimization phase. Parameters like RMSE and loss are calculated along with the processing of epochs and iterations to determine the quality of the trained model. The trained model should have less loss and a lower RMSE at the end of training to provide a high training accuracy factor.

The proposed LDW-CNN model outperforms the baseline models in terms of both recall and precision. The LDW-CNN model achieved a recall of 99.008%, significantly higher than that of CNN (96.85%) and MSVM (90.95%). This indicates that the proposed model has a stronger ability to correctly identify positive cases across the multiple diseases studied. Similarly, the LDW-CNN model achieved a precision of 97.054%, outperforming CNN (92.76%) and MSVM (81.00%), highlighting its effectiveness in minimizing false positives and providing more reliable disease predictions.

**Figure 7:**
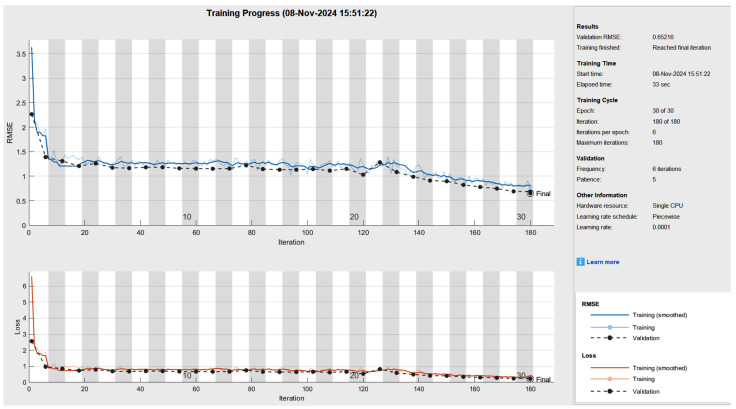
LDW-CNN training module.

These improvements are attributed to the integration of the linear discriminant analysis (LDA) and grey wolf optimization (GWO) techniques, which optimizes feature extraction and model hyperparameters, leading to better classification performance. Figure 7 shows the completed training process of the LDW-CNN architecture. The categories of heart, liver, and kidney disease are analyzed within this process for given test samples to determine the patient’s health situation.

**Figure 8:**
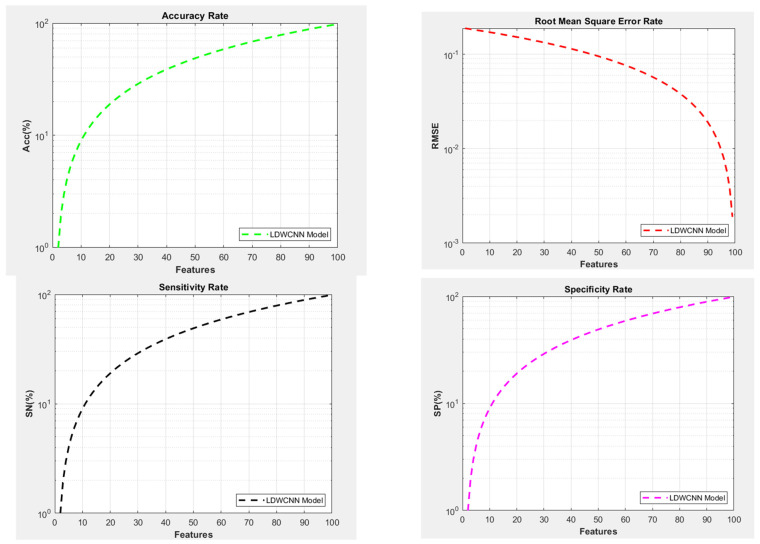
LDWgraphs of performance parameters.

Processing the data from the classification module and calculating the probability values for health status show the level of disease and the patient’s condition and help generate recommendations. The healthy sample will not receive any suggestions, but on the scale of healthy to critical, the proposed architecture has various recommendations, such as recommendations for exercise, visits to the doctor, etc. The performance of the LDW-CNN model is calculated using multiple parameters. These are based on the classification and generated recommendations for the test set. Performance metrics like accuracy, sensitivity, and specificity should be high for the model to demonstrate high performance, and RMSE should be lower to reduce errors. The accuracy of the proposed architecture is 98.19, the RMSE is 0.1878, sensitivity is 99.08, and specificity is 99.18. This demonstrates the high performance of the proposed architecture in classifying various diseases and recommending treatments. In Figure 8, the RMSE is depicted, which demonstrates high performance and is lower than that of the other trained models in comparison. RMSE is used to measure the difference between actual and predicted values, and it should be lower than other performance parameters, because as RMSE decreases, the model’s accuracy increases.

The proposed architecture shows an RMSE of 0.1878 in the test case. Accuracy is another factor that determines the performance of trained models. The higher the accuracy, the better the model is considered in terms of classification and recommendation. Figure 8 shows the proposed accuracy for the current test case, which is 98.19, with 100 feature sets on the X-axis. The terms “specificity” and “sensitivity” are used to evaluate the performance of a classifier on a given dataset. Specificity measures the rate of correctly classified negative samples compared to the quantity of actual negative samples in the dataset. The proposed LDW-CNN model (shown in Figure 8) has a high specificity rate of 99.18, which means it accurately evaluates negative samples in the testing set. On the other hand, sensitivity measures the actual positive rate, or the rate of correctly classified positive samples, compared to the quantity of actual positive samples in the dataset. The proposed LDW-CNN model showed a sensitivity of 99.08 for 100 feature sets at the X level in Figure 8. This demonstrates a higher rate of sensitivity compared to other executions.

### 4.2. Comparison of GWO-Based LDA/CNN Model with Existing Models

**Figure 9:**
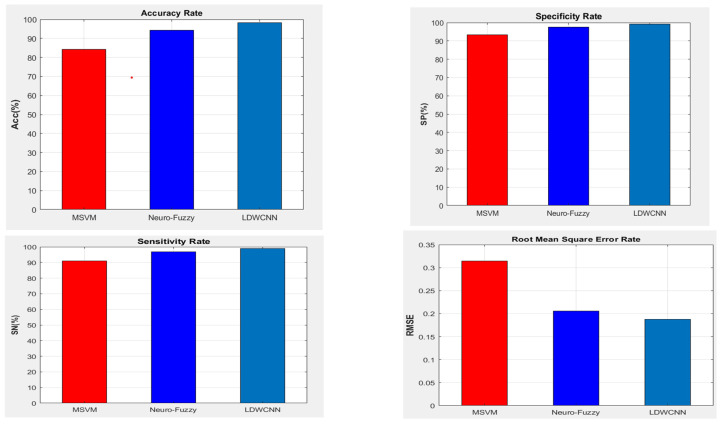
Comparison chart between the MSVM, neuro-fuzzy and LDWCNN models.

We experimented on a healthcare dataset consisting of 1032 patient reviews. The dataset was split into development and test data at a ratio of 75:25. The findings were evaluated using a 10-fold cross-validation approach. We used techniques like LDA, CNN, and GWO to design and assess the health recommendation system, including evaluation of specific details. We used the RMSE method to measure and identify the quality factor of the recommendation systems. Figure 9 presents a comparison chart demonstrating that the proposed classification and recommendation model achieves superior accuracy to the multi-classification MSVM and CNN models. The integration of LDA and GWO techniques demonstrates effectiveness and efficiency. Moreover, the LDW-CNN model significantly reduces computational time compared to existing methods. For large and complex datasets, deep learning-based healthcare recommender systems excel by adeptly identifying complex patterns and relationships within the data, resulting in enhanced performance. Conversely, health recommender systems utilizing multi-class classification methods are more appropriate for datasets with fewer variables and smaller dimensions. The grey wolf optimizer used in this model has the potential to improve performance compared to using default or manually tuned parameters. Although deep learning models often achieve higher accuracy, they require lengthier training periods and may have limited interpretability. Conversely, multi-class classification models offer greater interpretability but might not match the performance of deep learning-based models. Table 7 presents a comparison of the performance parameters for various classification algorithms.

Figure 9 compares existing multi-level (MSVM) and neuro-fuzzy approaches with the proposed LDW-CNN approach. The value of RMSE is compared with existing techniques to identify the enhancements provided by the optimized proposed architecture. The RMSE of the proposed model should be low, and the graph also shows that it is lower than that of the existing multi-level (MSVM) and neuro-fuzzy recommender systems. Comparing the accuracy of a trained model with other models is crucial to determining its performance with real-time samples and its efficiency in producing analysis reports for a specific execution. Higher accuracy indicates models of good quality. Figure 9 compares three models based on accuracy, where the proposed LDW-CNN model performs better than the others, with a high accuracy of 98.19%. The specificity rate shows how efficiently a model classifies negative samples compared to actual negative samples in the dataset. A high specificity rate ensures accurate evaluation of negative samples during testing, indicating better quality output by the model. The proposed LDW-CNN model showed a high specificity rate compared to existing techniques, ranging from 99.18% to 99.27%. The proposed LDW-CNN architecture showed enhanced results due to the optimal feature extraction and CNN architecture. It provides a better sensitivity rate of 99.08 in the above execution. We have incorporated both LDA, for feature reduction and extraction, and GWO, for parameter optimization; the classification model used in the healthcare recommendation system can benefit from improved efficiency, reduced overfitting, and better generalization. The integration of LDA and GWO can be performed iteratively, where LDA helps in feature transformation, and GWO optimizes the model parameters. This iterative process can lead to incremental improvements in the classification model’s performance over multiple iterations, and we obtained better accuracy. The various test cases are performed with different divisions of datasets within the proposed architecture to see the performance and calculations compared to datasets of other sizes, as shown in Table 8. Some samples of results of the prediction and recommendation phase are given in Table 9.

The LDW-CNN model’s high performance can be attributed to the combined strengths of LDA, GWO, and the CNN. LDA reduces dimensionality, allowing the model to focus on key discriminative features, while GWO optimizes both feature selection and CNN hyperparameters, enhancing accuracy and reducing overfitting. The CNN’s ability to capture complex patterns with ReLU activation further boosts classification performance, achieving an accuracy of 98.19%.

However, the model faces challenges in handling edge cases, such as rare diseases and overlapping symptoms. In these situations, the model may misclassify due to insufficient data or ambiguity in symptom patterns. Additionally, class imbalance in the dataset has introduced bias, leading to better predictions for more common diseases like heart disease, while underperforming on rarer conditions. To address these issues, future work will focus on more balanced datasets and techniques like resampling or weighted loss functions to improve fairness across disease categories.

### 4.3. Model Limitations and Instances of Underperformance

While the LDW-CNN model demonstrates high accuracy in classifying diseases, it is important to acknowledge certain limitations and instances of underperformance. The model’s performance is influenced by the quality of the input data. Issues such as missing values or inaccurate labels can lead to misclassifications. Ensuring thorough data pre-processing is essential for improving reliability. The datasets used often exhibit class imbalance, where some diseases are underrepresented. This can result in lower sensitivity for less common conditions, as the model may favor predictions for more frequent diseases. Despite achieving an accuracy of 98.19%, the model may struggle with atypical patient profiles or overlapping symptoms, leading to misclassifications. We will include examples of test cases where unexpected predictions occurred in the larger dataset. In terms of critical metrics, we evaluated the proposed research using accuracy, specificity, sensitivity, and root mean square error. However, while the LDW-CNN model has demonstrated strong performance on historical datasets, it is important to note that real-time data was not utilized in this study, which is a significant limitation. This limitation could affect the model’s adaptability in dynamic healthcare environments, where patient conditions may change rapidly. Without real-time data, the model’s ability to handle continuously evolving inputs is untested, which may impact its generalizability and responsiveness in real-world scenarios. Nevertheless, we are confident in the stability and performance of the model in a dynamic healthcare environment. We believe the proposed algorithm’s proven accuracy and robustness are superior to existing models and represent a significant advancement in healthcare recommendation systems. The strength of the LDW-CNN algorithm lies in its ability to detect complex data patterns that conventional machine learning methods may struggle to recognize.

## 5. Conclusions and Future Scope

Our study presents a multi-level decision-making framework for healthcare recommendation systems incorporating the LDW-CNN technique. We are confident that this deep learning-based approach will pave the way for more accurate and robust healthcare recommendation systems. The significant contributions of this study are as follows:

The proposed algorithm exhibits enhanced accuracy and robustness compared to existing models. Its superior performance is evident in its ability to deliver more precise results and maintain stability across various conditions, outperforming current methodologies regarding reliability and effectiveness. The LDW-CNN technique effectively mitigates issues such as information degradation and computational latency, resulting in enhanced class separability and operational efficiency compared to conventional CNN-based recommendation systems. Additionally, the GWO’s optimization capabilities refine the model’s accuracy and generalization, demonstrating its effectiveness in real-world healthcare scenarios. The proposed model can be adapted and tested on various medical datasets beyond disease detection, such as medical imaging and genomic data, to assess its versatility and robustness.

While the LDW-CNN model demonstrates high performance metrics, a significant limitation is the absence of real-time data integration. The effectiveness of the model in practical settings is uncertain without the use of ongoing patient data. Real-time data is crucial for dynamic patient monitoring, allowing timely adjustments to treatment plans and enhancing the model’s adaptability to changing health conditions. For successful clinical application, challenges such as interoperability with existing electronic health records, user-friendly interfaces for healthcare professionals, and data privacy concerns must be overcome. Future work should focus on validating the model with real-time clinical data to assess its performance and ensure that it generalizes well across diverse patient populations.

As we move forward, we are confident that integrating data from diverse sources, including electronic health records, wearable devices, and genetic information, will enable a more comprehensive understanding of patient health and better recommendations. We are also confident that we can create a recommendation system interface that can be accessed through web browsers and smartphones, making healthcare recommendations more accessible and convenient for patients. In future work, we aim to integrate real-time data to validate the model’s performance in live clinical settings, ensuring its robustness and accuracy in responding to real-time health conditions. The proposed model can provide immediate benefits in clinical settings when used with real-time diagnostic tools and healthcare systems, aiding in faster and more accurate decision-making.

## Figures and Tables

**Table 1:** Existing methods for healthcare recommendation systems that have a research gap.

References	Methodology	Objective	Performance Measures	Research Gap
Subiksha et al. [19]	Deep learning-based health analyzer system	To develop a deep learning model for personalized healthcare recommendations	Accuracy, Precision	Struggles with imbalanced datasets, leading to biased predictions in rare disease cases.
Sharma et al. [23]	Information retrieval approach for healthcare recommendation system	To improve information retrieval in healthcare systems for effective decision-making	Retrieval Accuracy, Latency	Needs integration of more disease features and a scalable architecture to handle large datasets.
Mudaliar et al. [20]	Machine learning-based health recommender system	To predict diseases and recommend treatments based on patient data	Accuracy, Sensitivity	Lack of advanced text mining techniques leads to the loss of important patient information.
Ambikai et al. [26]	PCA and SVM algorithm-based healthcare system	addresses key issues such as information quality, trustworthiness, authentication, and privacy concerns	Accuracy, Precision	Lack accurate prediction, personalized recommendations, and behavior-based insights,
Rana et al. [27]	Machine learning (ML)-based approach to generating healthcare predictions	To forecast disease occurrence based on patient data	Accuracy, Precision, F1-Score	Is limited by the small dataset size, leading to overfitting. More extensive patient data collection is needed for better generalization.
Yang et al. [28]	Cluster-based hierarchical approach for healthcare recommendations	To cluster patient data and provide personalized recommendations	Clustering Accuracy, Computation Time	Requires more data to improve the accuracy of clustering and recommendations. Handling large-scale data efficiently remains a challenge.
Sharma et al. [29]	Deep learning and neuro-fuzzy-based system for healthcare recommendations	To predict and evaluate the severity of diseases in real-time healthcare settings	Accuracy, Recall, Computational Efficiency	Struggles with real-time data processing and limited interpretability. Further work is needed on real-time data integration and explainability.

**Table 2:** Final set of hyperparameters.

Hyperparameter	Value	Rationale
Learning Rate	0.001	This learning rate offered a balance between training speed and convergence.
Filter Sizes	3 × 3	These filter sizes were selected to capture local patterns and features relevant to healthcare data, ensuring a fine-grained analysis of disease markers.
Dropout Rate	0.5	This dropout rate was used to prevent overfitting during training, ensuring that the model generalized well to unseen data.
Epochs	50	Early stopping was applied to avoid overfitting, and 50 epochs were found to be sufficient for optimal performance without sacrificing accuracy.
Optimizer	Adam	The Adam optimizer was chosen for its adaptive learning rate, improving model convergence for complex datasets.

**Table 3:** Dataset featuring a range of diseases [29].

Common Attributes	Heart Disease Attributes	Liver Disease Attributes	Kidney Disease Attributes
Age	Cp(Chest pain)	TB Total Bilirubin	Blood Pressure
Sex	Chol (Cholesterol)	DB Direct Bilirubin	ane—anemia
Blood pressure	Trestbps (resting blood pressure)	Alkphos Alkaline Phosphatase	albumin
ane—anemia	Restecg (resting ECG)	SgptAlamine Aminotransferase	sugar
albumin	Thalach (Maximum Heart rate)	Sgot Aspartate Aminotranferase	Red blood cells
Sugar	Slope (Slope of the peak exercise)	TP Total Proteins	Wc—white blood cells
Red blood cells	Ca (number of major vessels (0–3))	ALB Albumin	rc—red blood cell count
Wc—white blood cell	Painloc (Chest pain location)	A/G Ratio Albumin	Appet—appetite
rc—red blood cell count	Tharlest (resting heart rate)	Globulin Ratio	Cad—coronary artery disease

**Table 4:** Liver health status parameters [29].

Parameters	Range	Minimum	Maximum
Aspartate transaminase	0–35 IU/I.	10 units	40 units
Alanine transaminase	0–45 IU/I.	7 units	56 units

**Table 5:** Kidney health status parameters [29].

Parameter	Gender	Maximum	Minimum
Serum	Male	120	60
	Female	110	50
Potassium			
5.3 to 5.5 mmol/L	Medium		
5.3 to 5.5 mmol/L	High		
Above 6 mmol/L	Very High (Life-Threatening)		

**Table 6:** Heart health status parameters [29].

Parameter	Range	Risk of Disease
Blood Pressure		
BP 90/60 (Low) (mm Hg)	90/60	High
BP 120/80 (Normal)	120/80	Fit (No Disease)
BP 140/190 (High)	140/190	Very High
Cholesterol		
100 to 129 mg/dL	100 to 129 mg/dL	Fit (No Disease)
130 to 159 mg/dL	130 to 159 mg/dL	Border Line
160 to 189 mg/dL	160 to 189 mg/dL	High
190 mg/dL and above	190 mg/dL and above	Very High

**Table 7:** Comparison of the performance parameters.

Parameters	CNN	Multi-Level	LDW-CNN
Accuracy	94.28	84.3	98.19
RMSE	0.2057	0.31	0.18
Recall (Sensitivity)	96.85	90.95	99.0081
Precision	92.76	81.00	97.05
Specificity	97.43	93.35	99.18
F Score	92.76	81.0054	97.054

**Table 8:** Analysis of classification experiments across various parameters.

Training Set (%)	Test Set (%)	Accuracy	Specificity	Sensitivity	RMSE
60	40	97.99	97.09	98.02	0.1821
70	30	98.08	98.67	99.12	0.181
60	50	97.4	95.9	96.8	0.19
40	100	91.99	94.13	92.9	0.229
60	100	94.1	95.16	93.99	0.21
80	20	98.19	99.18	99.08	0.1878

**Table 9:** Outcomes of the prediction and recommendation phase.

S. No.	Patient Id	Level	Disease	Prediction
1	92	0.5	Heart Disease	Need to visit a doctor
2	121	0.5	Heart Disease	Need to visit a doctor
3	165	0.25	Heart Disease	Need normal exercise
4	254	0.5	Liver Disease	Need to visit a doctor
5	320	1	Liver Disease	Need to get hospitalized and have proper treatment
6	412	0	Liver Disease	Need normal exercise
7	500	1	Liver Disease	Need to get hospitalized and have proper treatment
8	545	0	Kidney Disease	Need normal exercise
9	610	>0	No Disease	Patient is normal
10	612	>0	No Disease	Patient is normal

## Data Availability

The datasets generated and/or analyzed during the original study are openly available: Heart Disease Dataset [37]; Chronic_Kidney_Disease Dataset [38]; ILPD (Indian Liver Patient Dataset) [39].

## References

[1] Ricci F., Rokach L., Shapira B. (2011). Introduction to Recommender Systems Handbook.

[2] Sánchez-Moreno D., López Batista V.F., Muñoz Vicente M.D., Sánchez Lázaro Á.L., Moreno-García M.N. (2024). Social Network Community Detection to Deal with Gray-Sheep and Cold-Start Problems in Music Recommender Systems. Information.

[3] Javaid M., Haleem A., Singh R.P., Ahmed M. (2024). Computer vision to enhance healthcare domain: An overview of features, implementation, and opportunities. Intell. Pharm..

[4] Al-Assaf K., Alzahmi W., Alshaikh R., Bahroun Z., Ahmed V. (2024). The Relative Importance of Key Factors for Integrating Enterprise Resource Planning (ERP) Systems and Performance Management Practices in the UAE Healthcare Sector. Big Data Cogn. Comput..

[5] Amini Gougeh R., Zilic Z. (2024). Systematic Review of IoT-Based Solutions for User Tracking: Towards Smarter Lifestyle, Wellness and Health Management. Sensors.

[6] Ogunleye B., Sharma H., Shobayo O. (2024). Sentiment Informed Sentence BERT-Ensemble Algorithm for Depression Detection. Big Data Cogn. Comput..

[7] Marinakis I., Karampidis K., Papadourakis G. (2024). Pulmonary Nodule Detection, Segmentation and Classification Using Deep Learning: A Comprehensive Literature Review. BioMedInformatics.

[8] Chinnasamy P., Wong W., Raja A.A., Khalaf O.I., Kiran A., Babu J.C. (2023). Health Recommendation System using Deep Learning-based Collaborative Filtering. Heliyon.

[9] Esmaeilzadeh P. (2024). Challenges and strategies for wide-scale artificial intelligence (AI) deployment in healthcare practices: A perspective for healthcare organizations. Artif. Intell. Med..

[10] Abughazalah M., Alsaggaf W., Saifuddin S., Sarhan S. (2024). Centralized vs. Decentralized Cloud Computing in Healthcare. Appl. Sci..

[11] Xiao F., Lai K.K., Lau C.K., Ram B. (2024). Robust Overbooking for No-Shows and Cancellations in Healthcare. Mathematics.

[12] Talha M.M., Khan H.U., Iqbal S., Alghobiri M., Iqbal T., Fayyaz M. (2023). Deep learning in news recommender systems: A comprehensive survey, challenges and future trends. Neurocomputing.

[13] Lee J.-Y., Lee S.Y. (2024). Development of an AI-Based Predictive Algorithm for Early Diagnosis of High-Risk Dementia Groups among the Elderly: Utilizing Health Lifelog Data. Healthcare.

[14] Mishra A., Tabassum N., Aggarwal A., Kim Y.-M., Khan F. (2024). Artificial Intelligence-Driven Analysis of Antimicrobial-Resistant and Biofilm-Forming Pathogens on Biotic and Abiotic Surfaces. Antibiotics.

[15] Gamil S., Zeng F., Alrifaey M., Asim M., Ahmad N. (2024). An Efficient AdaBoost Algorithm for Enhancing Skin Cancer Detection and Classification. Algorithms.

[16] Tefera M.A., Dehnaw A.M., Manie Y.C., Yao C.-K., Bogale S.D., Peng P.-C. (2024). Advanced Denoising and Meta-Learning Techniques for Enhancing Smart Health Monitoring Using Wearable Sensors. Future Internet.

[17] Martelli E., Capoccia L., Di Francesco M., Cavallo E., Pezzulla M.G., Giudice G., Bauleo A., Coppola G., Panagrosso M. (2024). Current Applications and Future Perspectives of Artificial and Biomimetic Intelligence in Vascular Surgery and Peripheral Artery Disease. Biomimetics.

[18] Yoo H., Chung K. (2018). Mining-based lifecare recommendation using peer-to-peer dataset and adaptive decision feedback. Peer--Peer Netw. Appl..

[19] Subiksha K. (2018). Improvement in analyzing healthcare systems using deep learning architecture. Proceedings of the 2018 4th International Conference on Computing Communication and Automation (ICCCA).

[20] Mudaliar V., Savaridaasan P., Garg S. (2019). Disease prediction and drug recommendation android application using data mining (virtual doctor). Int. J. Recent Technol. Eng..

[21] Sahoo A.K., Pradhan C., Barik R.K., Dubey H. (2019). Deepreco: Deep learning based health recommender system using collaborative filtering. Computation.

[22] Theodore Armand T.P., Mozumder M.A.I., Carole K.S., Deji-Oloruntoba O., Kim H.-C., Ajakwe S.O. (2024). ELIPF: Explicit Learning Framework for Pre-Emptive Forecasting, Early Detection and Curtailment of Idiopathic Pulmonary Fibrosis Disease. BioMedInformatics.

[23] Sharma M., Ahuja L., Bhattacharyya P., Sastry H., Marriboyina V., Sharma R. (2018). A Data Mining Approach Towards HealthCare Recommender System. Smart and Innovative Trends in Next Generation Computing Technologies NGCT 2017.

[24] Sanchez D., Melin P., Castillo O. (2017). A Grey Wolf Optimizer for Modular Granular Neural Networks for Human Recognition. Comput. Intell. Neurosci..

[25] He X., Qi G., Zhu Z., Li Y., Cong B., Bai L. (2023). Medical image segmentation method based on multi-feature interaction and fusion over cloud computing. Simul. Model. Pract. Theory.

[26] Ambika M., Latha K. Intelligence Based Recommender System for Healthcare: A Patient-Centered Framework. Proceedings of the 2nd International Conference on Advanced Theoretical Computer Applications.

[27] Rana C., Jain S.K. (2015). A study of the dynamic features of recommender systems. Artif. Intell. Rev..

[28] Yang D., Zhang J., Wang S., Zhang X.D. (2019). A Time-Aware CNN-Based Personalized Recommender System. Complexity.

[29] Sharma D., Aujla G.S., Bajaj R. (2021). “Deep neuro-fuzzy approach for risk and severity prediction using recommendation systems in connected health care”. Trans. Emerg. Telecommun. Technol..

[30] Miladinović A., Accardo A., Jarmolowska J., Marusic U., Ajčević M. (2024). Optimizing Real-Time MI-BCI Performance in Post-Stroke Patients: Impact of Time Window Duration on Classification Accuracy and Responsiveness. Sensors.

[31] Gao L., Wu S., Wongwasuratthakul P., Chen Z., Cai W., Li Q., Lin L.L. (2024). Label-Free Surface-Enhanced Raman Spectroscopy with Machine Learning for the Diagnosis of Thyroid Cancer by Using Fine-Needle Aspiration Liquid Samples. Biosensors.

[32] Avelar F.M., Lanza C.R.M., Bernardino S.S., Garcia-Junior M.A., Martins M.M., Carneiro M.G., de Azevedo V.A.C., Sabino-Silva R. (2024). Salivary Molecular Spectroscopy with Machine Learning Algorithms for a Diagnostic Triage for Amelogenesis Imperfecta. Int. J. Mol. Sci..

[33] Fu X., Guo D., Hou K., Zhu H., Chen W., Xu D. (2024). Fault Diagnosis of an Excitation System Using a Fuzzy Neural Network Optimized by a Novel Adaptive Grey Wolf Optimizer. Processes.

[34] Ragab M., Kateb F., Al-Rabia M.W., Hamed D., Althaqafi T., AL-Ghamdi A.S.A.-M. (2023). A Machine Learning Approach for Monitoring and Classifying Healthcare Data—A Case of Emergency Department of KSA Hospitals. Int. J. Environ. Res. Public Health.

[35] Kushimoto K., Obata Y., Yamada T., Kinoshita M., Akiyama K., Sawa T. (2024). Variational Mode Decomposition Analysis of Electroencephalograms during General Anesthesia: Using the Grey Wolf Optimizer to Determine Hyperparameters. Sensors.

[36] Akinyelu A.A., Zaccagna F., Grist J.T., Castelli M., Rundo L. (2022). Brain Tumor Diagnosis Using Machine Learning, Convolutional Neural Networks, Capsule Neural Networks and Vision Transformers, Applied to MRI: A Survey. J. Imaging.

[37] http://archive.ics.uci.edu/ml/datasets/heart+disease.

[38] http://archive.ics.uci.edu/ml/datasets/chronic_kidney_disease.

[39] https://archive.ics.uci.edu/ml/datasets/ILPD+(Indian+Liver+Patient+Dataset).

[40] Vuille-Lessard É., Rodrigues S.G., Berzigotti A. (2021). Noninvasive Detection of Clinically Significant Portal Hypertension in Compensated Advanced Chronic Liver Disease. Clin. Liver Dis..

[41] Hossain M.I., Hossain M.H., Khan M.A.R., Prity F.S., Fatema S., Ejaz M.S., Khan M.A.S. (2023). Heart disease prediction using distinct artificial intelligence techniques: Performance analysis and comparison. Iran J. Comput. Sci..

